# Serum Proteomic Signatures in Cervical Cancer: Current Status and Future Directions

**DOI:** 10.3390/cancers16091629

**Published:** 2024-04-24

**Authors:** Chaston Weaver, Alisha Nam, Caitlin Settle, Madelyn Overton, Maya Giddens, Katherine P. Richardson, Rachael Piver, David P. Mysona, Bunja Rungruang, Sharad Ghamande, Richard McIndoe, Sharad Purohit

**Affiliations:** 1 Center for Biotechnology and Genomic Medicine, Medical College of Georgia, Augusta University, Augusta, GA 30912, USA; chweaver@augusta.edu (C.W.); katrichardson@augusta.edu (K.P.R.); rpiver@augusta.edu (R.P.); dmysona@augusta.edu (D.P.M.); rmcindoe@augusta.edu (R.M.); 2 Department of Undergraduate Health Professions, College of Allied Health Sciences, Augusta University, Augusta, GA 30912, USA; anam@augusta.edu (A.N.); casettle@augusta.edu (C.S.); moverton@augusta.edu (M.O.); magiddens@augusta.edu (M.G.); 3 Department of Obstetrics and Gynecology, Medical College of Georgia, Augusta University, Augusta, GA 30912, USA; brungruang@augusta.edu (B.R.); sghamande@augusta.edu (S.G.)

**Keywords:** cervical cancer, inflammation, biomarkers, proteomics, bioinformatics, artificial intelligence

## Abstract

**Simple Summary:**

In this literature review, we delve into the emerging prospects of of leveraging proteomic signatures and additional molecular data to enhance the clinical care and management of cervical cancer (CC) patients. By critical evaluation of existing research findings and elucidating recent advancements, we aim to provide an understanding of how proteomic signatures and molecular information on diagnostic, prognostic, and therapeutic strategies for CC. This review underscores the importance of integrating proteomic analysis into clinical practice, offering insights into its role in enhancing personalized treatment approaches and improving patient outcomes in the realm of cervical cancer management. Through a critical examination of current literature, we highlight the promising avenues for leveraging proteomic data to address the multifaceted challenges associated with CC diagnosis, treatment, and monitoring.

**Abstract:**

In 2020, the World Health Organization (WHO) reported 604,000 new diagnoses of cervical cancer (CC) worldwide, and over 300,000 CC-related fatalities. The vast majority of CC cases are caused by persistent human papillomavirus (HPV) infections. HPV-related CC incidence and mortality rates have declined worldwide because of increased HPV vaccination and CC screening with the Papanicolaou test (PAP test). Despite these significant improvements, developing countries face difficulty implementing these programs, while developed nations are challenged with identifying HPV-independent cases. Molecular and proteomic information obtained from blood or tumor samples have a strong potential to provide information on malignancy progression and response to therapy in CC. There is a large amount of published biomarker data related to CC available but the extensive validation required by the FDA approval for clinical use is lacking. The ability of researchers to use the big data obtained from clinical studies and to draw meaningful relationships from these data are two obstacles that must be overcome for implementation into clinical practice. We report on identified multimarker panels of serum proteomic studies in CC for the past 5 years, the potential for modern computational biology efforts, and the utilization of nationwide biobanks to bridge the gap between multivariate protein signature development and the prediction of clinically relevant CC patient outcomes.

## 1. Introduction

Cervical cancer (CC) is the fourth most frequently diagnosed cancer in women worldwide [1]. Squamous cell carcinoma (SCC) accounts for over 75–80% of all cases of cervical cancer across the globe [2]. The remaining 20–25% of cases are adenocarcinomas and other rare forms with mixed features [2]. Human papillomavirus (HPV) is integral to the development of SCC [3]. Of the 20 different cancer-related HPVs, HPV-16 and 18 account for 70% of cervical cancer cases [4]. HPV is subtyped into low and high-risk variants, and the high-risk variants (hrHPV) are known to cause the malignant transformation of cervical epithelial cells (CECs) [5].

On a global scale, decreasing trends in CC burden, measured by incidence, death, and disability adjusted life years (DALYs), were noted from 1990–2019 [6]. Regions and countries may be characterized and assessed on CC burden according to their sociodemographic index (SDI), which encompasses per capita income, education, and fertility rates [7]. Developing countries and regions such as sub-Saharan Africa rank much lower in SDI and have higher CC burden and mortality rates due to deficits in screening services and education on preventive measures [6]. For these areas with a lower SDI, the improvement of public health initiatives towards increasing education and access to screening and HPV vaccination is of utmost importance in reducing the CC burden.

Areas classified as having a high SDI (North America, Finland, Australia) are more likely to have nationwide vaccination (HPV-vaccination) and screening programs (Papanicolaou (Pap) tests), contributing significantly to the global decreases in CC burden by early detection [6]. However, the rates of advanced-stage CC diagnosis are increasing in the United States at a rate of 1.3% per year [8]. An unfortunate consequence of the effectiveness of HPV vaccination and screening programs is an increased number of missed HPV-independent CC diagnoses. Several studies have found that HPV-independent CC cases are frequently diagnosed at later stages with reduced disease-free survival (DFS) and overall survival (OS) [9]. Racial disparities exist for CC as mortality (attributed to CC) is 1.5 times higher among African Americans than Caucasian women [8]. Compared to Caucasian women, the incidence rate of cervical cancer is 32% higher among Hispanic women. This incidence rate is 78% in Caucasian women from Puerto Rico [10], suggesting the absence of effective public health initiatives. Given these disparities among vulnerable populations, there is a growing interest in exploring the utility of molecular markers which could complement current methods of screening, diagnosis, and individualized treatment options [11]. The focus has also shifted towards improvements in the efficiency of population screening in efforts to address the disparities gap.

**Figure 1:**
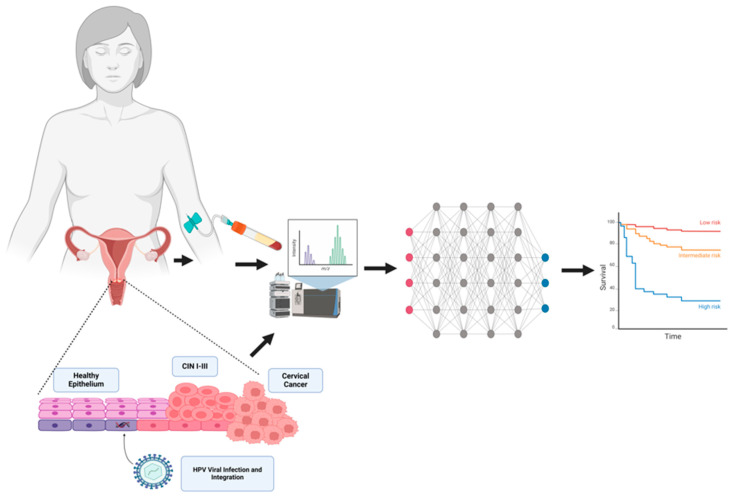
The overview of the identifying proteomic signature and their use in patient management in cervical cancer. Proteomic signatures can be identified and developed from blood or the pre-cancerous and cancerous lesions by employing immuno-assays or mass-spectrometry. The proteomic data from a sizeable population are then fed into a bioinformatic or artificial pipeline to reduce the complexity. The output then can be used for monitoring therapeutic benefits in the form of survival or to design novel therapies or treatments with lowered toxicities for improved survival. This figure was created on BioRender.

The PCR-based assay for hrHPV detection has largely replaced cytological sampling in many high-income countries, yet these tests still lack specificity and result in over referrals for gynecological assessment and unnecessary treatment [12] (PMID: 34551656). In this respect, the development of molecular marker-based technologies provides an avenue for improved specificity. As the majority of CC cases are linked to hrHPV infection, there is strong interest between protein alteration, viral replication, and cellular proliferation [12,13]. Studying protein expression helps to summarize the consequences of molecular events such as mutations and altered gene expression in the pathogenesis of cancer [14] (Figure 1).These protein biomarkers may also serve as targets for precision therapy development, and clinical monitoring [14] (Figure 1). MS-based proteomic analysis is central to the discovery phase of protein marker studies, but this process requires skilled expertise, which makes it an unreasonable option for direct use in low- and middle-income countries [12].

Following improvements to public health initiatives and screening, developing countries may greatly benefit from the creation of low-cost assays of validated marker panels. Still, there remains much work to be done in the identification and validation of these protein markers to develop cost-effective assays that can be implemented in areas of the greatest need. As such, we undertook the present literature review to examine serum proteomic markers associated with diagnosis and prognosis in women with cervical cancer. We also highlight future directives and the implementation of machine learning algorithms in light of the increasing addition of serum proteomic data to relevant databases. The development of novel therapies for cervical cancer is essential given the poor outcomes in those with advanced disease; however, there are limited data on the use of biomarkers to measure treatment response, monitoring, clearance, and metabolism, in comparison to other malignancies. Despite many of the advantages of other liquid-based proteomic analyses, the focus of this review will center on serum-based sample proteomics and their application in the diagnosis, prognosis, and predictive outcomes in CC patient cohorts.

## 2. Proteomic Changes in Cervical Cancer Cells

Proteomic analyses offer insight into the dynamic environment of protein expression and post-translational modifications within malignant cells [15]. This analysis allows for protein identification, characterization, expression rates, abundance, stability, and interaction with associated metabolic pathways [16,17]. The most commonly used techniques include mass spectrometry and immunoassays, and these modalities may incorporate other quantitative and high-throughput techniques [14,18,19].

The most comprehensive molecular characterization of CC to date was performed by The Cancer Genome Atlas (TCGA) Research Network in 2017. Their findings describe the molecular events of CC tumor development through a process of DNA mutations, copy number alterations (CNAs), and epigenetic changes which drive genomic instability [20]. Proteomic analysis using reverse phase protein array (RPPA) revealed three distinct clusters of differentially expressed proteins associated with hormone–receptor, EMT, and PI3K-AKT pathway dysregulation. Among the dysregulated proteins, the EMT cluster was associated with the worst outcomes for overall survival (OS) [20]. In addition to TCGA, several studies have identified protein biomarkers for cervical cancer which may be associated with clinical metrics of interest associated with diagnosis, prognosis, and therapeutic targeting, which are shown in Table 1 and Table 2.

Characterizations of the CC proteome have been accomplished through previous analyses of CC cell lines, fresh tissue, or bodily fluids such as urine and blood [14]. Many of these studies revealed single protein biomarkers, which provide useful information for patient diagnosis and prognosis; however, panels or signatures of proteins may be more effective markers for the detection and prediction of cancer outcomes [32]. A recent study by Ji et al. (2023) incorporated protein expression data from The Cancer Proteome Atlas (TCPA) and TCGA databases to find an expression signature associated with OS [33]. Prognosis-related proteins (PRPs) were identified using a univariate Cox regression analysis to discover an optimized apoptosis-related signature of the three proteins, BCL2, SMAD3, and 4EBP1-pT70 [33]. All three proteins are involved in the regulatory mechanisms associated with apoptosis, the dysregulation of which is a hallmark of cancer [34]. Many of the proteins involved in apoptotic pathway dysregulation are multi-functional in their roles and interactions, which has prompted the need for continued improvements in high-throughput technologies and large-scale database accessibility to reveal important associations between biomarkers and disease processes [33]. As technological advancements continue to be made in the identification phase of biomarker discovery pipelines, there remains much work to be done in narrowing the gap between detection and clinical utilization.

Despite improvements to proteomic analyses over recent decades, there are still many barriers to the clinical application of cellular proteomic workflows. Some of these barriers include the dynamic nature of protein expression within the cell, biochemical and structural differences of proteins within different tissue samples, complex protein–ligand interactions, and technical limitations of analytic techniques requiring highly trained personnel [35]. In light of this, blood samples present a promising source for the clinical application of proteomics due to their minimally invasive collection process and the reservoir of diverse and dynamic protein content within [36]. The large amount of proteomic information obtained from serum and plasma, which can approach an analyte concentration on the order of 10^10^, makes analyzing large numbers of samples challenging [37]. Only recently have certain advances to mass spectrometry (MS) instrumentation and improved computational methodologies paved a way for the practical application of protein biomarkers in clinics [37].

Improvements to multiplexing workflows, such as multiple reaction monitoring (MRM), and the addition of immunoaffinity antipeptide antibodies to MS are two examples of preanalytical developments which have allowed the processing of serum samples using the conventional methods of protein analysis [38,39]. As the exploration into the proteomic environment continues, the application of these new data for CC patients relies on clearly defined clinical goals, consistency in experimental procedures, and the extensive external validation of new biomarkers [40]. Cellular proteomics has been integral in the identification of novel drug targets and pathways that can be modulated to increase patient survival and lower drug toxicity [41]. However, cellular proteomics have limitations in patient monitoring for survival and therapeutic response as tumor samples and tumor proximal fluids require more invasive collection methods compared to blood-based samples [35,42]. Secreted proteins found in the serum are of particular interest as they are influential in the coordination between nonadjacent tissues, and ultimately regulate complex processes that result in human diseases such as cancer [43]. Antibody microarray has been successfully used to identify protein signatures from serum samples which provide prognostic data in breast [44] and prostate [45] cancer, and can be a potential tool for clinical use. For patient monitoring, a collection of serum proteins may be a better alternative. To date, there are still very few serum protein biomarkers which have gained FDA-clearance for use in clinical settings [46].

## 3. Serum Protein Markers in CC

Much of the current literature investigating the CC proteome involves single markers, which have poor sensitivity and specificity for the prognosis and diagnosis of cancer compared to a multimarker approach [32]. To investigate the current state of proteomic serum biomarkers in CC, a literature search was conducted in Medline via PubMed, using the keywords “cervical cancer” AND “protein biomarkers” AND “serum.” The search was restricted to articles published between 2018 and 2023. The inclusion criteria were restricted to articles with more than one protein biomarker, metrics of analysis associated with diagnostic, prognostic, and predictive capabilities (hazard ratio (HR), area under the receiver operating characteristic (AUROC), sensitivity (SN), specificity (SP), and appropriate proteomic analysis techniques for serum-based biomarkers such as ELISA, MS, and other iterations of these techniques. The search results yielded 140 articles, 12 of which matched our inclusion criteria; the findings are summarized in Table 1 and Table 2. Serum proteins associated with clinical metrics of interest include those associated with diagnostic, prognostic, and predictive therapeutic response in CC patients. Serum samples have historically been chosen as the standard for clinical chemistry tests versus plasma samples [47]. Serum samples differ from plasma samples in that cellular components are easily separated, they have greater stability, and they lack clotting factors which allows for an easier application of proteomic techniques for analysis [47].

### 3.1. Proteomic Studies with Diagnostic Potential

The most widely studied protein among CC patients is the squamous cell carcinoma antigen (SCC-Ag). In addition to CC, the SCC-Ag is also used as a biomarker in HPV-derived SCC of the head, neck, lung, and esophagus [48]. The most attractive feature of the SCC-Ag is its association with clinical outcomes, therapeutic response, and tumor resistance [24,48]. The SCC-Ag by itself has limitations in sensitivity and specificity in predicting early-stage CC; however, Zhang et al. (2022) found that when combined with CXC motif chemokine 10 (CXCL10), the discriminatory power improved for CC versus healthy controls compared to either biomarker alone (AUCCXCL10 = 0.775, AUCSCCAg = 0.793 vs. AUCCXCL10 + SCC-Ag = 0.877, *p* < 0.05) [24] (Table 1). The role of inflammatory biomarkers in CC extends to other chemokines which may be paired with the SCC-Ag, such as serum CXCL8 and CXCR2.

Zhang et al. (2023) reported an improvement in AUC values for distinguishing healthy controls from CC patients with this upregulated protein signature (SCC-AG, CXCL8, CXCR2) compared to the three individual proteins alone [25] (Table 1). In a pilot study from Thailand (*n* = 31), a six protein panel showed improved sensitivity and specificity for differentiating healthy controls from CC patients as well as early vs. late stage patient subgroups [21] (Table 1). The differential expression of these proteins was associated with healthy controls from CC patients with an AUC of 0.933 (84.6% sensitivity and 87.5% specificity) [21] (Table 1). In addition to the limitations of the small sample size, the methods utilized to extract and detect the proteins reported in this study are time consuming and would be difficult to implement in routine clinical practice. Despite being underpowered, these results are a positive step in the clinical management of CC and thus, it is important to validate this signature in larger patient cohorts.

### 3.2. Proteomic Signatures for Prognosis in CC

There are a number of progression-related proteins (PRPs) that have been identified in serum samples from patients with CC, including the vascular endothelial growth factor (VEGF) protein. The VEGF protein and its receptors have been implicated as a major pathway associated with tumor angiogenesis and targeted VEGF inhibition constitutes an FDA-approved treatment approach in CC, renal cell carcinoma (RCC), and hepatocellular carcinoma (HCC) patients [49,50]. Sawada et al. (2019) found elevated serum concentrations of VEGF-A and VEGFR-2 to be associated with significantly worse OS compared to low concentrations [28] (Table 2). High VEGF-A concentration was also associated with a bulky tumor size (>40 mm) and pelvic lymph node involvement (PLNI), which may help to guide chemotherapy and radiotherapy treatment options [28]. Serum PRPs associated with the immunopathogenesis of CC may also predict patient prognosis. These proteins include components of the complement lectin system and involve the mannan-binding lectin (MBL)-associated serine proteases (MASPs), MASP-1 and MASP-2, and the MBL-associated protein (MAp-19) [51]. Maestri et al. (2018) found that a high expression of these three proteins was significantly associated with poor survival and relapse, as the increased activation of this complement system may be associated with cervical basement membrane disruption and tumor invasion [27] (Table 2).

There is a limited amount of evidence available for serum biomarkers of interest specific to adenocarcinoma of the cervix (ACC) compared to SCC, despite increasing trends in incidence and poor survival outcomes in ACC [52,53]. Liu et al. (2022) developed a nomogram based on clinical variables and an upregulated serum protein marker panel to predict OS and PFS with moderate accuracy [31] (Table 2). Serum markers including the carcinoembryonic antigen (CEA), neuron-specific enolase (NSE), and human chorionic gonadotropin (HCG-ß) were independent prognostic factors for CC patients [31] (Table 2). When combined with clinical factors including FIGO stage and para-aortic lymph node involvement (PALN), this algorithm more accurately predicted survival (OS and PFS) compared to FIGO classification alone (HR (OS): 2.281, 2.166, 2.584; HR (PFS): 2.116, 2.217, 2.478) [31]. Risk stratification systems such as this nomogram allow for the finer stratification of patients than the standard staging; in turn, this allows for the strategic management of treatment and predictive response for targeted therapy.

Predictive response to radiation therapy (RT), brachytherapy, surgery, and treatment planning on an individual basis will likely be obtained through the improved sensitivity of identifiable biomarkers [54]. The SCC-Ag, in conjunction with the serum biomarker apolipoprotein C-II (ApoC-II) [55], have shown association with OS, progression free survival (PFS), distant metastasis free survival (DMFS), as well as pelvic progression-free survival (PPFS) for Japanese patients receiving radiation therapy (RT) (Table 2) [30]. Surgical prognosis was evaluated by Chen et al. (2019), who identified the differential expression of three distinct peptide fragments (TKT, FGA, & ApoA-1) among pre- and post-surgical patients, but this study was limited due to the small number of clinical samples [26] (Table 2). Purohit et al. (2020) previously established a senescence-associated secretory phenotype (SASP)-based eight protein signature, which associated this signature with an improved response to brachytherapy [29] (Table 2). When patients were subgrouped into moderate and high cellular senescence, brachytherapy showed a significant survival benefit (5 year disease-specific survival (DSS)) compared to no brachytherapy and low cellular senescence [29] (Table 2).

One of the greatest difficulties faced by the current state of proteomic analysis is deconvoluting big data at an early point in proteomic workflows. In addition, inconsistencies within clinical studies have slowed the progression of the clinical utilization of biomarker data, as evidenced by the wide variation of analytical and experimental techniques exhibited in the studies in Table 1 and Table 2. Addressing the current issues of complex data management and streamlining clinical study designs have the potential to shorten the gap between continued protein biomarker identification and clinical implementation.

## 4. Future Directions in CC Serum Proteomics

There is a severely limited amount of serum proteomic data currently available in the way of multimarker panels, as evidenced by the collection of studies in Table 1 and Table 2. One of the major limitations consistent throughout the available data is that these studies do not approach the necessary scale in sample size and control to approximate population-based interpretations. Still, some of the largest genome-wide association studies (GWASs) to date report sample sizes approaching only a few thousand [56]. One of the potential solutions to the slow development of biomarker pipelines in cancer research is the utilization of biobanks [57]. With the increasing development of biobanks in several countries [58,59,60], hundreds of thousands of blood samples from patients across multiple demographic variables become available for use in developing and validating proteomic data associated with cancer diagnostics, prognostics, and predictive response to treatment. In addition to being a rich resource for genomic data, biobanks may also provide insights into environmental cofactors to proteogenomic dysregulation which promote disease proliferation [56]. One of the major limitations in utilizing the wealth of information that biobanks provide is that most traditional statistical methods of regression are inadequate for analysis due to the high degree of dimensionality of the data [61]. The generation of big data, whether from biobank resources or selected patient cohorts, requires more powerful tools for analysis. Artificial intelligence (AI)-based techniques such as machine and deep learning algorithms have been utilized to find statistically accurate and meaningful relationships between molecular data and patient outcomes [62].

In the age of the increasing expansion of proteomic techniques capable of exploring the human proteome more deeply and generating big data, the need for methods of analysis beyond conventional bioinformatic methods has become apparent. The function of machine learning (ML) has been evident for over a decade in various parts of the MS-based proteomic workflow, including the use of support vector machines, and gradient boosting for peptide identification and MS/MS spectrum prediction [63,64]. Deciphering LC-MS/MS data has typically relied on the use of proteomic spectral libraries such as PRIDE [65]. This has resulted in some limitations regarding MS analysis validation due to specific experimental constraints under which the spectra were catalogued, resulting in spectral peak matches that may be made in error or completely erroneous altogether [66,67]. Data-driven modeling through ML overcomes this by training on previous sets of observational data, allowing for complex relation fitting in a nonspecific and unbiased manner [68]. Smaller sized data may be appropriately handled by classical ML algorithms, but feature engineering becomes more complex as the size of the data is enlarged [67]. The proper execution of feature engineering through ML requires accurate input feature representation of the data prior to analysis, which is subject to human error and requires domain expertise [67]. Deep learning (DL) models of ML have the ability to solve these complex problems in large proteomic data sets due to end-to-end learning strategies [69], which apply specialized layers of the neural network to optimize feature construction and improve overall model performance without the need for self-assignment [67,70]. Despite two successful transitions of proteomic signatures into clinical practice in areas other than gynecological cancer [71], there remains space for the increased implementation of ML models, which may alleviate some of the current issues with signature validation and efficient assay design.

The use of AI in CC has been investigated in areas such as automated cytological analysis [72], colposcopy imaging prognostics [73], post-surgical complications [74], and radiation toxicity [75]. Still, there remains a limited utilization of ML regarding biomarker discovery and validation in CC, unlike other gynecological cancers such as ovarian cancer (OC). Several studies have used ML algorithms for OC cytoreduction prognosis utilizing serum proteins. In a study by Kawakami et al. (2019), multiple supervised ML algorithms were compared to assess the accuracy of residual tumor prediction following surgery [76]. In their analysis, the random forest (RF) classifier predicted clinical stage with an accuracy of 69% and an AUC of 0.76, and ensemble classifiers, as a whole, outperformed traditional regression analysis in separating benign and cancerous tumors [76]. Another study by Enshaei et al. (2014) used an artificial neural network (ANN) algorithm for surgical prediction with an accuracy of 77% and an AUC of 0.73, and was also able to predict OS more accurately than logistic regression [77]. In addition to surgical outcomes, Mysona et al. (2019) developed a serous high-grade ovarian cancer (SHOC) score to predict PFS, which implemented the elastic net ML algorithm to create a comprehensive multivariate score based on clinical and serum data [78]. In another study, a ML risk assessment score was able to predict ovarian cancer recurrence in patients following chemotherapy treatment [79]. The score utilized the elastic net ML algorithm to combine multiple clinical parameters and serum proteomic data to predict time-to-recurrence (TTR) and time-to-death (TTD), as well as PFS and OS in both low and high-risk groups [79]. The use of ML in OC provides some useful building blocks upon which proteomic studies in CC can build. Improvement in this area may lead to the increased validation of established CC serum biomarkers as well as improved efficacy, which are required for prospective trials and eventually FDA approval [80].

As strategies and analytical techniques continue to be developed on the back-end of proteomic panel identification and clinical association, much work is still required in the improvements to experimental techniques and quantification methods [40,81]. Many of these challenges will require improvements to quality control, sample standardization in processing and handling, and the implementation of consistent validation methods [81]. As many of these challenges continue to be overcome, future efforts towards leveraging advanced bioinformatic tools and artificial intelligence will likely play an influential role in the clinical utilization of proteomic signature data.

## 5. Conclusions

Improvements in the ability to screen the general population for CC, group patients for treatment stratification, and predict response and recurrent rates have the potential to diminish the physical and emotional burden on patients as well as the economic burden on women and society. Recent developments in high-throughput proteomic technologies have led to large-scale data generation, yet much of these data have not been translated to the clinical setting. Instead of low-resolution single markers, a panel of markers may provide an opportunity for the integration of ML algorithms to identify complex relationships between proteins in CC patient survival and prognosis. With the increasing use of ML algorithms within clinical studies, researchers will be better able to generate more clinically useful data, which are required for larger validation studies in randomized clinical trials. While a large proportion of this wealth of biomarker data goes unutilized, the proper validation of signature data along with current prevention programs may allow clinicians to achieve the shared goal of earlier diagnosis, prognosis, and patient management.

## Figures and Tables

**Table 1:** Available diagnostic serum proteomic signatures for clinical management and treatment of CC patients.

Proteins	Metric	Samples vs. Control	Histological Sample Type	Measure	Outcome	Ref.
** A1AT, PYCR2, TTR, APOA1, VDBP, MMRN1	AUC: 0.933 (SN: 84.6%, SP: 87.5%)	31 vs. 16	SCC: 22, ACC: 8, HCC: 1	Diagnostic	HPV + Normal control vs. CC (early stage)	[21]
** P1GF, F1T1	AUC: 0.8493 (SN: 70.45%, SP: 92.11%	62 vs. 20	CIN: 18, SCC: 31, ACC: 13	Diagnostic	Normal vs. CC	[22]
** ANT3-1, FBLN1-2	AUC: 0.6885 (SN: 76.92%, SP: 70.00%)	284 vs. 75	HSIL/CIN II/III: 88, SCC: 121	Diagnostic	Normal vs. CC	[23]
** CXCL10, SCC-Ag	AUC: 0.877	264 vs. 81	CIN: 75, SCC: 189	Diagnostic	Normal vs. CC	[24]
** CXCL8, CXCR2, SCC-Ag	AUC: 0.847 (SN: 85.71%, SP: 70.00%)	100 vs. 30	CIN I-III: 30, SCC: 70	Diagnostic	Normal vs. CC	[25]

** Bivariate/multivariate analysis, SN: sensitivity, SP: specificity, HSIL: high-grade squamous intraepithelial lesion, CIN: cervical intraepithelial neoplasia.

**Table 2:** List of proteomic studies showing prognostic potential for early prediction of outcomes in cervical cancers.

Proteins	Metric	Samples vs. Control	Histological Sample Type	Measure	Outcome	Ref.
TKT, FGA, APOA1	AUC = 0.8463, 0.8038, 0.7641	67 vs. 50	SCC: 29, ACC: 10 (pre-surgery)	Prognostic	Pre- vs. Post-operative surgical prognosis	[26]
MASP-2, MASP-1, MAp-19	^†^ SN: 71.3%, SP: 63.2%	292 vs. 52	CIN II/III: 214, CC: 78	Prognostic	Poor survival and Disease Relapse	[27]
VEGF-A, VEGFR-2	HR: 3.42, 6.37	107 vs. N/A	SCC: 80, ACC: 23, ASC: 3, SmCC: 1	Prognostic	Overall Survival	[28]
** CRP, GRO, LEPTIN, MIG, MMP1, SCCA, SAA, sIL2Rα	HR: 1.89, 2.07, 0.61, 1.85, 1.69, 3.55, 1.60, NA	565 vs. N/A	SCC: 565	Prognostic	DSS	[29]
^1^ SCC-Ag, ^2^ ApoC-II	^1^ HR: 1.186, 1.185, 1.260 ^2^ HR: 0.606	142 vs. N/A	SCC: 142	Predictive	(Pre-RT) ^1^ PFS, OS, DMFS ^2^ PPFS	[30]
CEA, NSE, HCG-ß	^3^ HR: 2.281, 2.166, 2.584 ^4^ HR: 2.116, 2.217, 2.478	295 vs. N/A	ACC: 295	Prognostic	^3^ OS, ^4^ PFS	[31]

** Bivariate/multivariate analysis, SN: sensitivity, SP: specificity, ^†^: Limited metric data available: SN/SP provided only for MASP-2, ^1,2,3,4^ denote metric (AUC, sensitivity/specificity, hazards ratio) association with individual proteins or outcomes. DSS: disease-specific survival, RT: radiation therapy, PFS: progression-free survival, DMFS: distant metastasis-free survival, PPFS: pelvic progression-free survival, ASC: adenosquamous carcinoma, SmCC: small-cell carcinoma. N/A: Not available.
